# Supplementary material for: IMI-driver: Integrating multi-level gene networks and multi-omics for cancer driver gene identification
Source: PLoS Comput Biol. 2024 Aug 26;20(8):e1012389. doi: 10.1371/journal.pcbi.1012389 (PMC11379397; doi:10.1371/journal.pcbi.1012389)
Supplement: S1 Text — (DOCX) [file pcbi.1012389.s001.docx]

Supplemental Materials for

IMI-driver: integrating multi-level gene networks and multi-omics for cancer driver gene identification

PeiTing Shi^1#^, JunMin Han^1#^, YingHao Zhang^1^, GuanPu Li^1^, Xionghui Zhou^1,2*^

^1^Hubei Key Laboratory of Agricultural Bioinformatics, College of Informatics, Huazhong Agricultural University, Wuhan, 430070 People’s Republic of China

^2^Key Laboratory of Smart Farming for Agricultural Animals, Ministry of Agriculture and Rural Affairs, People’s Republic of China

#This authors contribute equally to this work.

*****Correspondence: Correspondence should be addressed to X. Z. ([zhouxionghui@mail.hzau.edu.cn](mailto:zhouxionghui@mail.hzau.edu.cn); zhouxionghui6@gmail.com)

Prediction of novel cancer driver genes

Genes predicted as key genes in multiple cancer types are more likely to be new driver genes. Based on this hypothesis, we performed pan-cancer analysis on the predicted driver genes of all the cancer types to discover potential new driver genes.

We combined the predictions of different cancers to explore the common drivers of cancers by the following steps:

Step 1: Tag cancer-specific candidate genes. We tagged the top 100 predicted genes in each cancer as candidate genes.

Step 2: Construct candidate gene sets. We constructed a candidate gene set by concatenating all cancer-specific candidate genes.

Step 3: Count the number of predictions. We counted the frequency of each gene that appeared on the driver gene list of each cancer type.
