# Supplementary material for: IMI-driver: Integrating multi-level gene networks and multi-omics for cancer driver gene identification
Source: PLoS Comput Biol. 2024 Aug 26;20(8):e1012389. doi: 10.1371/journal.pcbi.1012389 (PMC11379397; doi:10.1371/journal.pcbi.1012389)
Supplement: S2 Text — (DOCX) [file pcbi.1012389.s002.docx]

Supplemental Materials for

IMI-driver: integrating multi-level gene networks and multi-omics for cancer driver gene identification

PeiTing Shi^1#^, JunMin Han^1#^, YingHao Zhang^1^, GuanPu Li^1^, Xionghui Zhou^1,2*^

^1^Hubei Key Laboratory of Agricultural Bioinformatics, College of Informatics, Huazhong Agricultural University, Wuhan, 430070 People’s Republic of China

^2^Key Laboratory of Smart Farming for Agricultural Animals, Ministry of Agriculture and Rural Affairs, People’s Republic of China

#This authors contribute equally to this work.

*****Correspondence: Correspondence should be addressed to X. Z. ([zhouxionghui@mail.hzau.edu.cn](mailto:zhouxionghui@mail.hzau.edu.cn); zhouxionghui6@gmail.com)

Stability evaluation of IMI-driver

In our approach, we designate the Top n (n = 100) genes predicted by IMI-driver as driver genes. To assess whether the setting of top n would impact the performance of the model, we varied n by setting it to different thresholds (n = 100, 200, 300, 400, 500). We evaluated the performance of our method at different thresholds using two methods: (1) randomly shuffling class labels, retraining our model, and comparing the cross-validation results on the real class label dataset with those on the randomly labeled dataset. (2) comparing the performance of our model with the best-performing model among all models (MCC of IMI-driver - the highest MCC of the other models).
