# Supplementary material for: IMI-driver: Integrating multi-level gene networks and multi-omics for cancer driver gene identification
Source: PLoS Comput Biol. 2024 Aug 26;20(8):e1012389. doi: 10.1371/journal.pcbi.1012389 (PMC11379397; doi:10.1371/journal.pcbi.1012389)
Supplement: S3 Text — (DOC) [file pcbi.1012389.s003.doc]

Supplemental Materials for

IMI-driver: integrating multi-level gene networks and multi-omics for cancer driver gene identification

PeiTing Shi^1#^, JunMin Han^1#^, YingHao Zhang^1^, GuanPu Li^1^, Xionghui Zhou^1,2*^

^1^Hubei Key Laboratory of Agricultural Bioinformatics, College of Informatics, Huazhong Agricultural University, Wuhan, 430070 People’s Republic of China

^2^Key Laboratory of Smart Farming for Agricultural Animals, Ministry of Agriculture and Rural Affairs, People’s Republic of China

#This authors contribute equally to this work.

*****Correspondence: Correspondence should be addressed to X. Z. ([zhouxionghui@mail.hzau.edu.cn](mailto:zhouxionghui@mail.hzau.edu.cn); zhouxionghui6@gmail.com)

Data collection

We obtained multi-omics data (gene expression, miRNA expression, somatic mutation, methylation data and clinical data) of 29 cancer types (see Supplementary Table 3 for more details) from The Cancer Genome Atlas (TCGA) via UCSC Xena (July 28, 2022) [1]. For the gene expression data and mutation data, the high-level processed version (i.e. level 3) were used directly. In this context, the data have already been processed (by UCSC Xena) to use genes as the basic units. For example, gene expression values have been processed to reflect the value for each gene, and somatic mutation data have been processed to indicate whether a somatic mutation exists for each gene. For the DNA methylation value, we focused on DNA methylation sites within promoter regions. Given that each gene can have multiple methylation sites, we selected all sites within the promoter region of each gene. We computed the median methylation level of these sites to represent the overall methylation level of each gene.

The human protein-protein interaction (PPI) data were downloaded from the STRING database v.11.0 [2]. The KEGG pathway data were retrieved from KEGG [3]. The transcription factor-target network was collected from TRRUST [4]. We also downloaded 45 biological traits from DORGE [5].

To evaluate the IMI-Driver, an unbiased and comprehensive set of known cancer genes is needed. Unfortunately, there is no such gold standard set of cancer genes, and each cancer gene set is biased toward particular features or study methods. In this work, we used nine cancer driver datasets (see Supplementary Table 2 for more details) to validate our approach, such as Cancer Gene Census (CGC; Tier 1; January 2019) [6], CGCpointMut, 20/20 Rule [7], HCD [8], OncoGene [9], CTAT, MouseMut [10], ConsistentSet and IntOGen [11], where IntOGen is a tumor-specific dataset. We selected 17 cancers that were shared between IntOGen and TCGA data when this data set was used to validate IMI-driver.

**Supplementary References**

1. MJ Goldman, B Craft, M Hastie, K Repečka, F McDade, A Kamath. et al. Visualizing and interpreting cancer genomics data via the Xena platform. *Nat Biotechnol*. 2020; 38: 675–678.
2. D.D Szklarczyk, AL Gable, KC Nastou, D Lyon, R Kirsch, S Pyysalo et al. The STRING database in 2021: customizable protein–protein networks, and functional characterization of user-uploaded gene/measurement sets. *Nucleic Acids Res*. 2021; 49: D605–D612.
3. Klukas,C. and Schreiber,F. Dynamic exploration and editing of KEGG pathway diagrams. *Bioinformatics*. 2007; 23: 344–350.
4. H Han, JW Cho, S Lee, A Yun, H Kim, D Bae. TRRUST v2: an expanded reference database of human and mouse transcriptional regulatory interactions. *Nucleic Acids Res.* 2018; 46: D380–D386.
5. J Lyu, JJ Li, J Su, F Peng, YE Chen, X Ge. et al. DORGE: Discovery of Oncogenes and tumoR suppressor genes using Genetic and Epigenetic features. *Science Advances*. 2020; 6: eaba6784.
6. Z Sondka, S Bamford, CG Cole, SA Ward, I Dunham, SA Forbes. The COSMIC Cancer Gene Census: describing genetic dysfunction across all human cancers. *Nat Rev Cancer*. 2018; 18: 696–705.
7. Vogelstein, B., Papadopoulos, N., Velculescu, V. E., Zhou, S., Diaz Jr, L. A., & Kinzler, K. W. Cancer Genome Landscapes. *Science*. 2013; 339: 1546–1558.
8. Tamborero, D., Gonzalez-Perez, A., Perez-Llamas, C., Deu-Pons, J., Kandoth, C., Reimand, J. Comprehensive identification of mutational cancer driver genes across 12 tumor types. *Sci Rep*. 2013; 3: 2650.
9. Y Liu, J Sun, M Zhao. ONGene: A literature-based database for human oncogenes. J Genet Genomics. 2017; 44: 119–121.
10. Kim, E., Hwang, S., Kim, H., Shim, H., Kang, B., Yang, S.,et. al. MouseNet v2: a database of gene networks for studying the laboratory mouse and eight other model vertebrates. *Nucleic Acids Res*. 2016; 44: D848-854.
11. Gonzalez-Perez, A., Perez-Llamas, C., Deu-Pons, J., Tamborero, D., Schroeder, M. P., Jene-Sanz, A. *et. al*. IntOGen-mutations identifies cancer drivers across tumor types. *Nat Methods*. 2013; 10: 1081–1082.
