# Supplementary material for: IMI-driver: Integrating multi-level gene networks and multi-omics for cancer driver gene identification
Source: PLoS Comput Biol. 2024 Aug 26;20(8):e1012389. doi: 10.1371/journal.pcbi.1012389 (PMC11379397; doi:10.1371/journal.pcbi.1012389)
Supplement: S4 Text — (DOCX) [file pcbi.1012389.s004.docx]

Supplemental Materials for

IMI-driver: integrating multi-level gene networks and multi-omics for cancer driver gene identification

PeiTing Shi^1#^, JunMin Han^1#^, YingHao Zhang^1^, GuanPu Li^1^, Xionghui Zhou^1,2*^

^1^Hubei Key Laboratory of Agricultural Bioinformatics, College of Informatics, Huazhong Agricultural University, Wuhan, 430070 People’s Republic of China

^2^Key Laboratory of Smart Farming for Agricultural Animals, Ministry of Agriculture and Rural Affairs, People’s Republic of China

#This authors contribute equally to this work.

*****Correspondence: Correspondence should be addressed to X. Z. ([zhouxionghui@mail.hzau.edu.cn](mailto:zhouxionghui@mail.hzau.edu.cn); zhouxionghui6@gmail.com)

The pipeline of constructing common networks

Adopting the same strategy of previous work, the high-confidence PPIs from STRING [1], with interaction scores greater than 800 were kept to construct the PPI network.

We used 200 human pathways from KEGG to construct the pathway similarity network [2]. Each gene $i$ was represented by a 200-dimensional binary vector, where each dimension indicated whether the gene was present in a corresponding pathway. The gene co-occurrence relationship of two genes $i$ and $j$ was calculated as the cosine of the angle between their vectors, given by

$r_{ij}^{(3)}=\frac{p_{i}\cdot p_{j}}{\left| p_{i} \right|\left| p_{j} \right|}$ (1)

where $p_{i}$ and $p_{j}$ were the vectors corresponding to the genes $i$ and $j$, respectively.

All the TF-target pairs from TRRUST [3] were obtained to construct the transcriptional regulatory network.

**Supplementary References**

1. D.D Szklarczyk, AL Gable, KC Nastou, D Lyon, R Kirsch, S Pyysalo et al. The STRING database in 2021: customizable protein–protein networks, and functional characterization of user-uploaded gene/measurement sets. *Nucleic Acids Res*. 2021; 49: D605–D612.
2. Y Chen, J Hao, W Jiang, T He, X Zhang, T Jiang, R Jiang. Identifying potential cancer driver genes by genomic data integration. *Sci Rep*. 2013; 3: 3538.
3. H Han, JW Cho, S Lee, A Yun, H Kim, D Bae. TRRUST v2: an expanded reference database of human and mouse transcriptional regulatory interactions. *Nucleic Acids Res.* 2018; 46: D380–D386.
