# Supplementary material for: IMI-driver: Integrating multi-level gene networks and multi-omics for cancer driver gene identification
Source: PLoS Comput Biol. 2024 Aug 26;20(8):e1012389. doi: 10.1371/journal.pcbi.1012389 (PMC11379397; doi:10.1371/journal.pcbi.1012389)
Supplement: S5 Text — (DOCX) [file pcbi.1012389.s005.docx]

Supplemental Materials for

IMI-driver: integrating multi-level gene networks and multi-omics for cancer driver gene identification

PeiTing Shi^1#^, JunMin Han^1#^, YingHao Zhang^1^, GuanPu Li^1^, Xionghui Zhou^1,2*^

^1^Hubei Key Laboratory of Agricultural Bioinformatics, College of Informatics, Huazhong Agricultural University, Wuhan, 430070 People’s Republic of China

^2^Key Laboratory of Smart Farming for Agricultural Animals, Ministry of Agriculture and Rural Affairs, People’s Republic of China

#This authors contribute equally to this work.

*****Correspondence: Correspondence should be addressed to X. Z. ([zhouxionghui@mail.hzau.edu.cn](mailto:zhouxionghui@mail.hzau.edu.cn); zhouxionghui6@gmail.com)

Description of assessment criteria

Traditional supervised learning model performance is primarily evaluated by accuracy, A the Receiver Operating Characteristic Curve (AUROC), recall, and precision. Receiver Operating Characteristic (ROC) and precision are also commonly used as performance indices for driver prediction models. However, ROC and precision can be deceptive and may overstate the results, especially on unbalanced datasets. Instead, the Matthews Correlation Coefficient (MCC) is a better evaluation metric to assess imbalanced data sets [1]. In this work, it was selected as the primary performance metric. All the evaluation metrics for model performance were shown as follows.

$Recall=\frac{TP}{TP+FN}$ (1)

$Precision=\frac{TP}{TP+FP}$ (2)

$F1=\frac{2*Precision*Recall}{Precision+Recall}$ (3)

$MCC=\frac{TP*TN-FP*FN}{\sqrt{(TP+FP)(TP+FN)(TN+FP)(TN+FN)}}$ (4)

Most current cancer driver gene prediction algorithms use only the top N potential driver genes to evaluate their performances. However, this approach does not measure the overall performance of the model. Therefore, we assess the power of each prediction model through a non-parametric test - the Kolmogorov–Smirnov (KS) test. Compared to the ranking of all genes predicted by each method, a smaller p-value can be obtained if the ranking of all driver genes is higher. Thus, we utilize the p-values from the KS test to evaluate the performance of various features (or networks) in predicting driver genes.

**Supplementary References**

1. Colaprico, A., Olsen, C., Bailey, M. H., Odom, G. J., Terkelsen, T., Silva, T. C. Interpreting pathways to discover cancer driver genes with Moonlight. *Nat Commun*. 2020; 11: 69.
