# Supplementary material for: IMI-driver: Integrating multi-level gene networks and multi-omics for cancer driver gene identification
Source: PLoS Comput Biol. 2024 Aug 26;20(8):e1012389. doi: 10.1371/journal.pcbi.1012389 (PMC11379397; doi:10.1371/journal.pcbi.1012389)
Supplement: S6 Text — (DOCX) [file pcbi.1012389.s006.docx]

Supplemental Materials for

IMI-driver: integrating multi-level gene networks and multi-omics for cancer driver gene identification

PeiTing Shi^1#^, JunMin Han^1#^, YingHao Zhang^1^, GuanPu Li^1^, Xionghui Zhou^1,2*^

^1^Hubei Key Laboratory of Agricultural Bioinformatics, College of Informatics, Huazhong Agricultural University, Wuhan, 430070 People’s Republic of China

^2^Key Laboratory of Smart Farming for Agricultural Animals, Ministry of Agriculture and Rural Affairs, People’s Republic of China

#This authors contribute equally to this work.

*****Correspondence: Correspondence should be addressed to X. Z. ([zhouxionghui@mail.hzau.edu.cn](mailto:zhouxionghui@mail.hzau.edu.cn); zhouxionghui6@gmail.com)

The pipeline of predicting model

Genes predicted as key genes in multiple cancer types are more likely to be new driver genes. Based on this hypothesis, we performed pan-cancer analysis on the predicted driver genes of all the cancer types to discover potential new driver genes. In this analysis, IMI-driver was constructed by using the IntOGen database [1] as the benchmark, since IntOGen integrates different driver prediction methods and provides a tumor-specific driver gene list with rich cancer-specificity information.

We combined the predictions of different cancers to explore the common drivers of cancers by the following steps:

Step 1: Labeling tags. We labeled all the genes using a known driver gene database.

Step 2: Data splits. We randomly divided all genes into K folds, with K-2 folds assigned to the training set and the remaining 2 folds assigned to the validation and test set respectively. Each fold of the data was used as a test set exactly once in K-fold cross-validation.

Step 3: Addressing sample imbalance. We generated new samples using the SMOTE algorithm to balance the positive and negative samples in the training set.

Step 4: Training of the classifier. we trained the classifier on the training set, used the validation set to identify the best hyperparameters for the classifier, and applied the classifier to the test set. Importantly, the training set, validation set, and test set were disjoint to ensure that samples in the test sets were never used during training and hyperparameter tuning.

Step 5: Prediction and evaluation. We pooled the prediction scores of all genes and identified potential cancer driver genes based on these scores. We evaluated the performance of the classifier using metrics such as MCC, AUROC, F1, and precision.

**Supplementary References**

1. Gonzalez-Perez, A., Perez-Llamas, C., Deu-Pons, J., Tamborero, D., Schroeder, M. P., Jene-Sanz, A. *et. al*. IntOGen-mutations identifies cancer drivers across tumor types. *Nat Methods*. 2013; 10: 1081–1082.
