# Supplementary figures and images for: IMI-driver: Integrating multi-level gene networks and multi-omics for cancer driver gene identification
Source: PLoS Comput Biol. 2024 Aug 26;20(8):e1012389. doi: 10.1371/journal.pcbi.1012389 (PMC11379397; doi:10.1371/journal.pcbi.1012389)

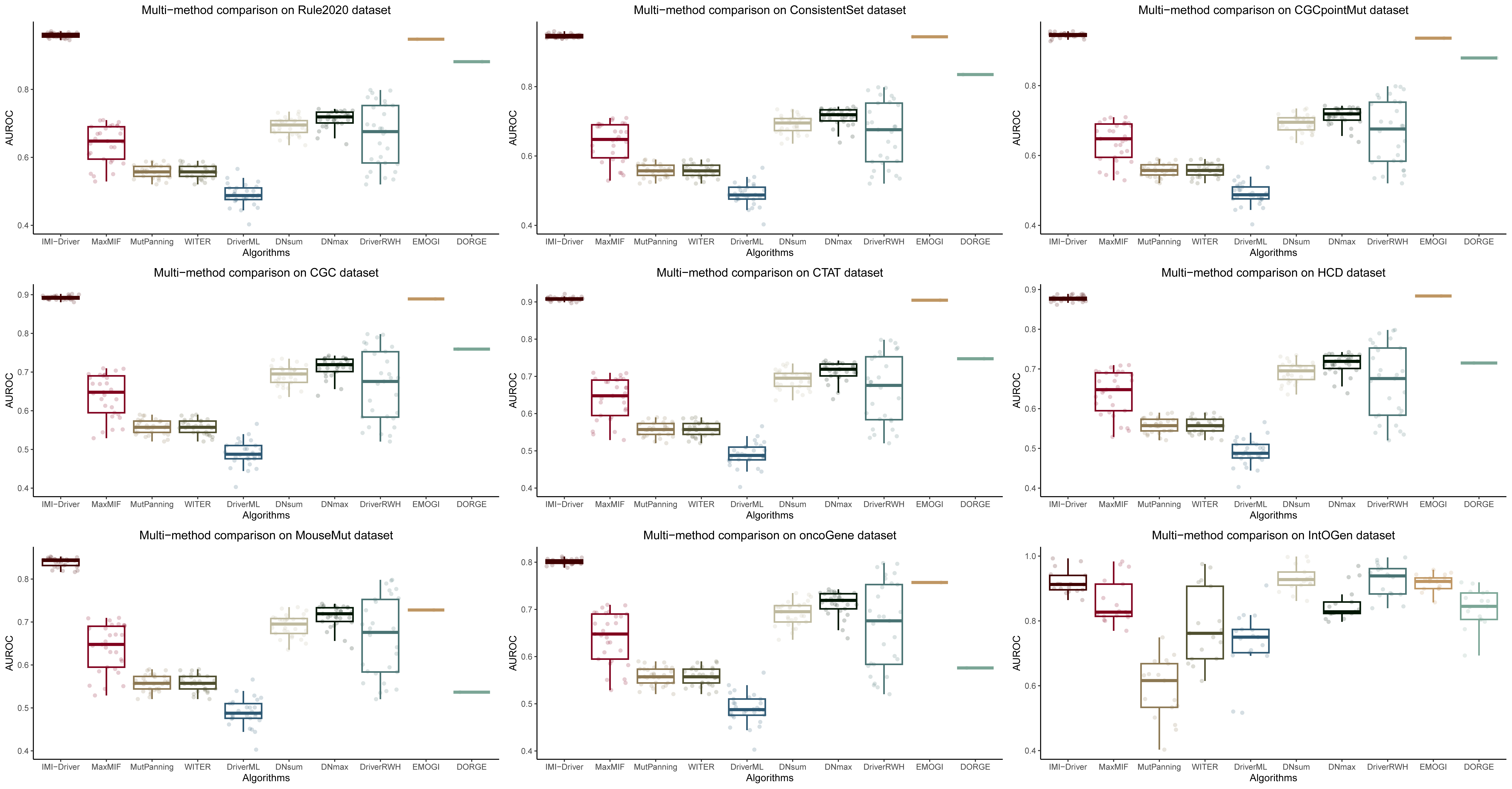

Supplement: S1 Fig — We compared the classification performance of IMI-driver with nine other methods across nine gold-standard datasets. Each subplot shows the classification performance comparison (AUROC) of the 10 methods on one benchmark dataset. (TIF) [file pcbi.1012389.s008.tif]

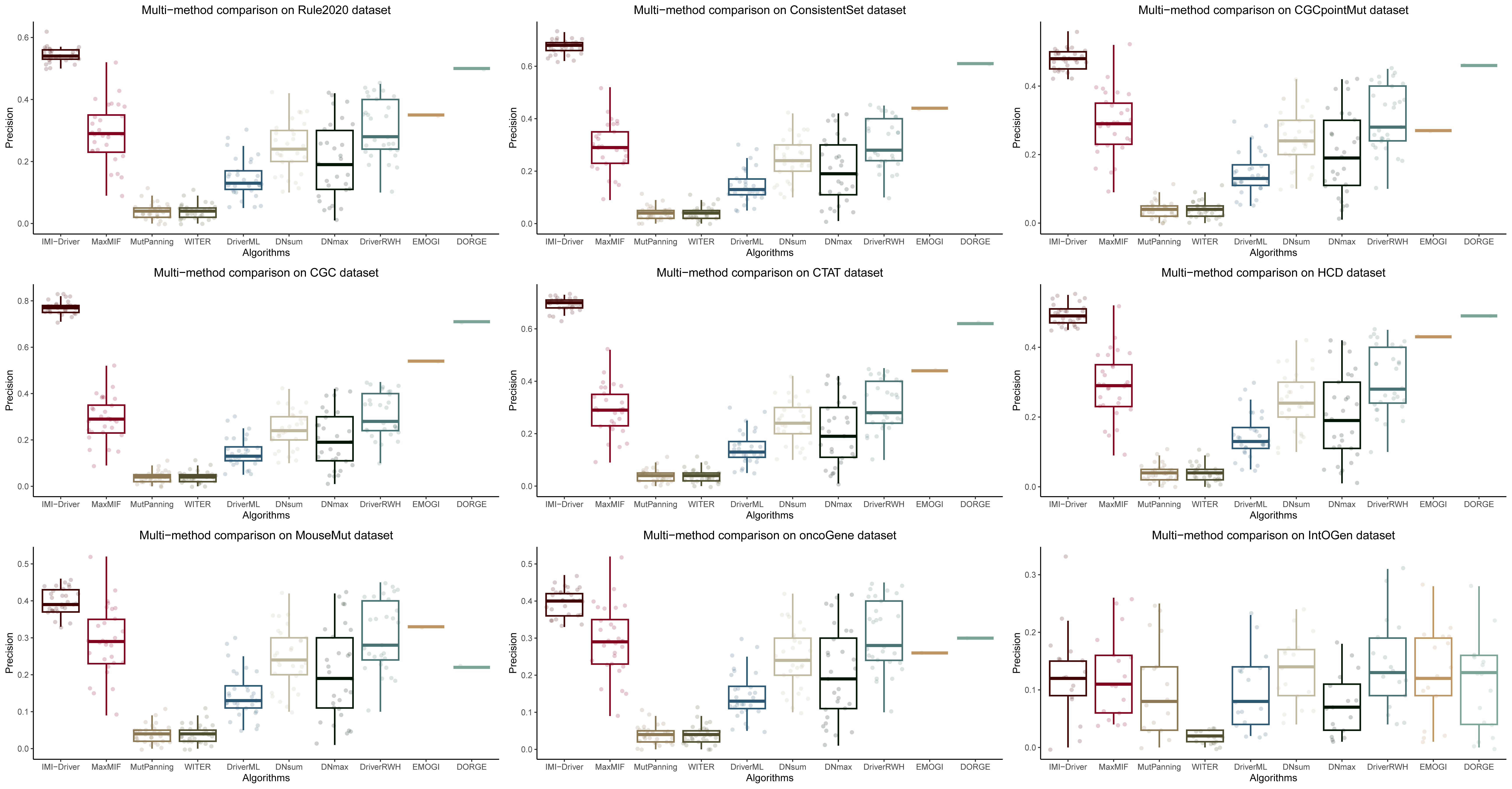

Supplement: S2 Fig — We compared the classification performance of IMI-driver with nine other methods across nine gold-standard datasets. Each subplot shows the classification performance comparison (Precision) of the 10 methods on one benchmark dataset. (TIF) [file pcbi.1012389.s009.tif]

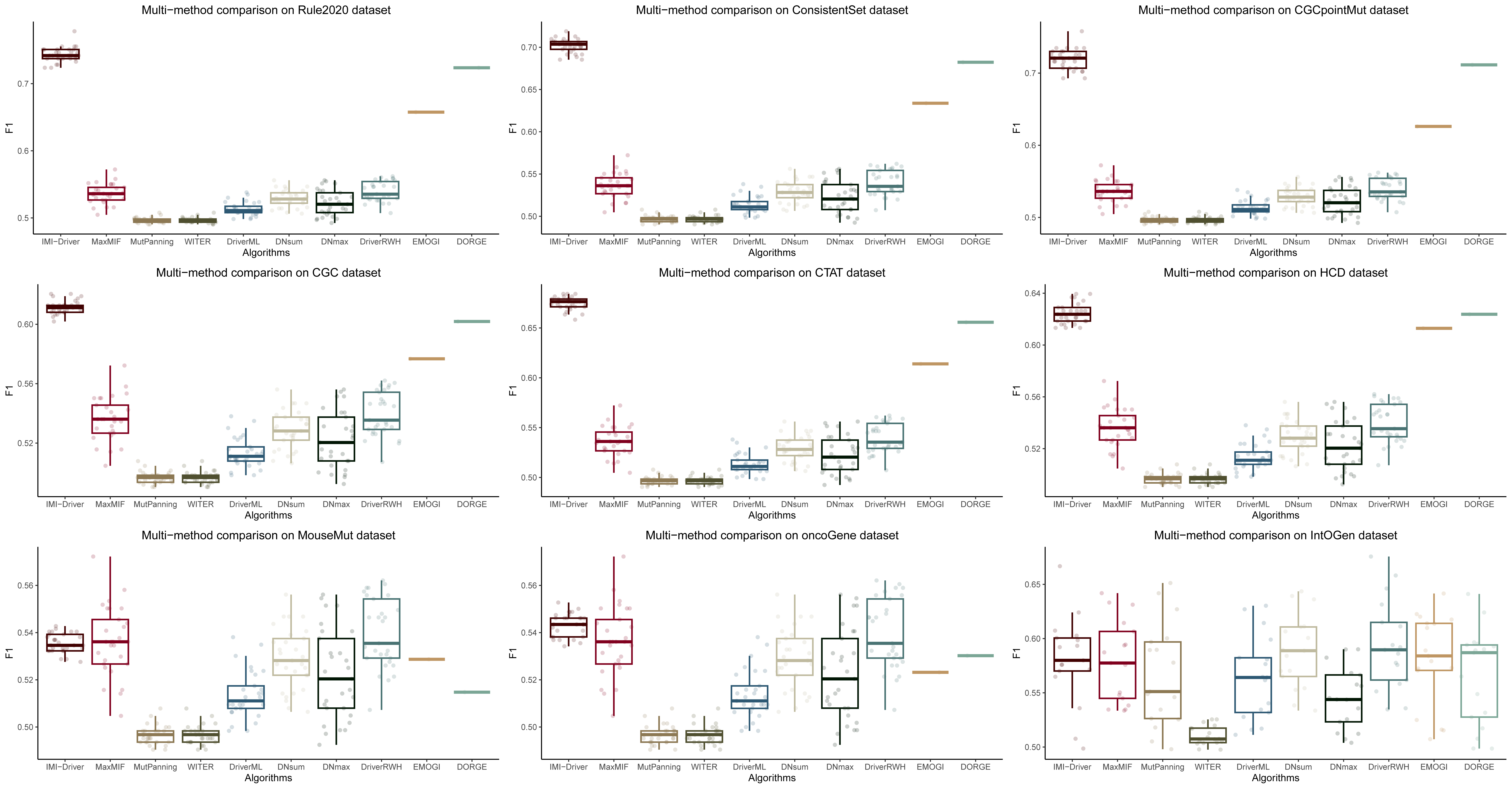

Supplement: S3 Fig — We compared the classification performance of IMI-driver with nine other methods across nine gold-standard datasets. Each subplot shows the classification performance comparison (F1-score) of the 10 methods on one benchmark dataset. (TIF) [file pcbi.1012389.s010.tif]

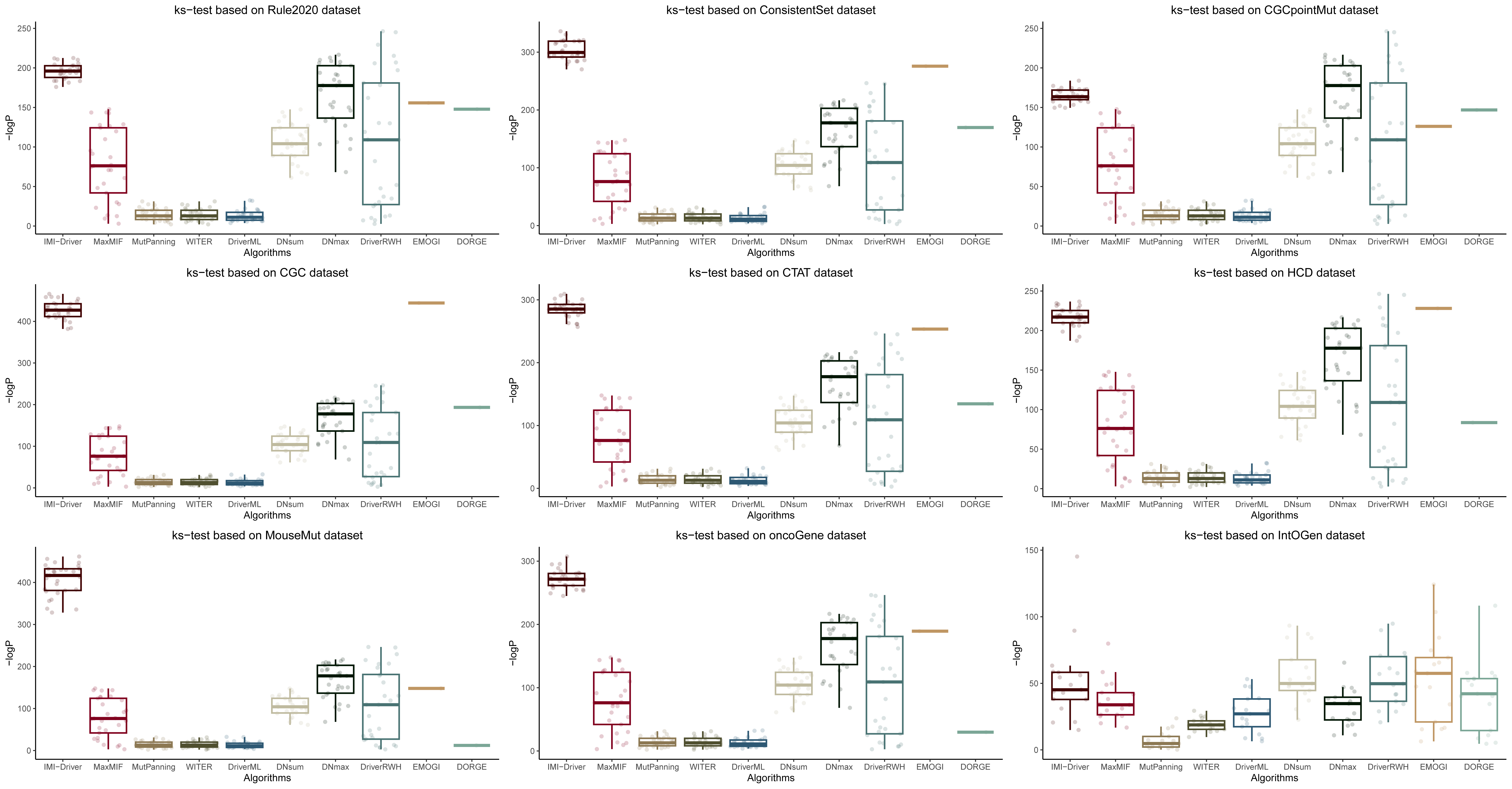

Supplement: S4 Fig — We compared the classification performance of IMI-driver with nine other methods across nine gold-standard datasets. Each subplot shows the classification performance comparison (KS test p-value) of the 10 methods on one benchmark dataset. (TIF) [file pcbi.1012389.s011.tif]

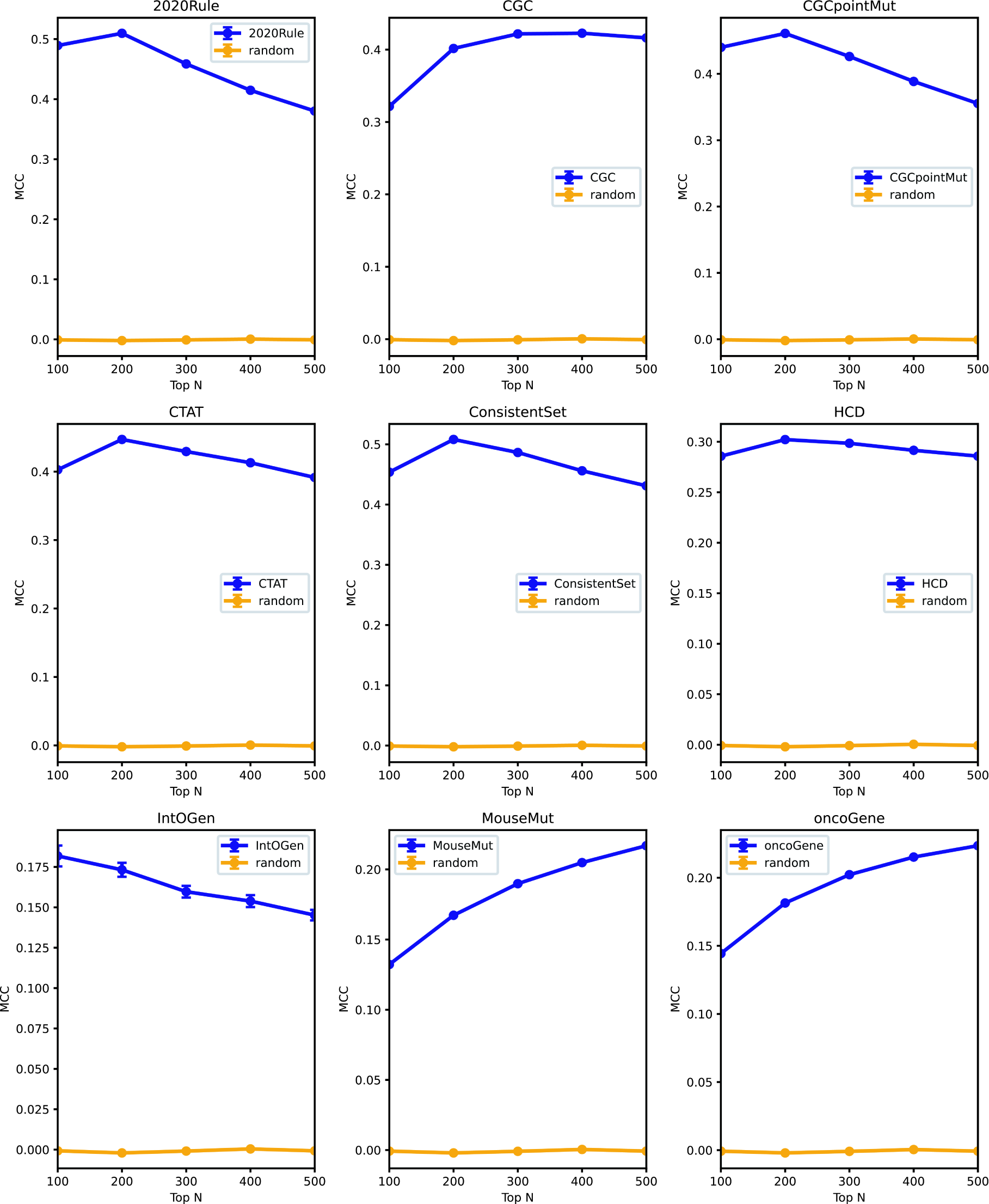

Supplement: S5 Fig — The models were compared across 9 benchmark datasets, with the x-axis representing the models at different thresholds (n) and the y-axis depicting the corresponding results (MCC). (TIF) [file pcbi.1012389.s012.tif]

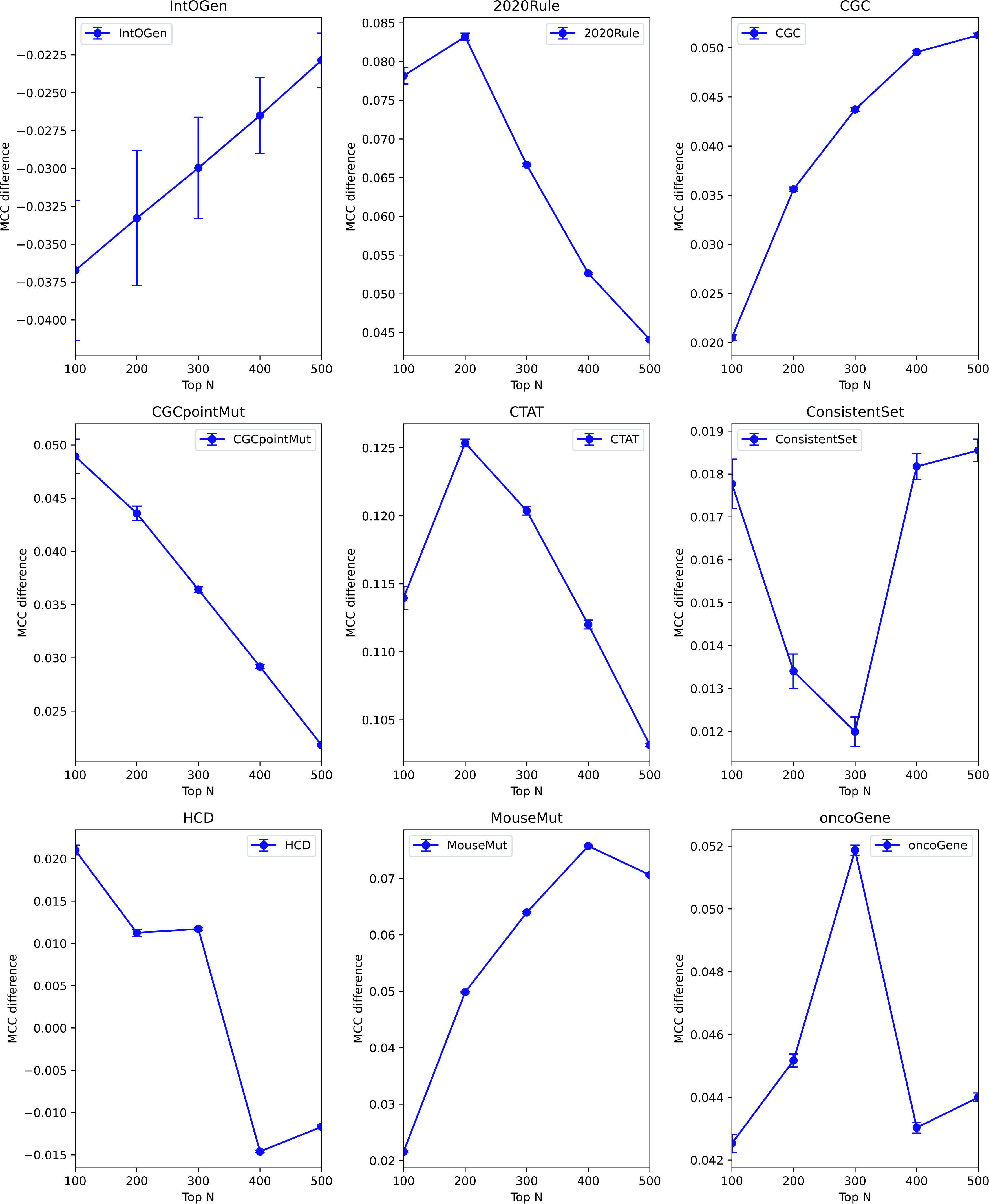

Supplement: S6 Fig — The models were compared across 9 benchmark datasets, with the x-axis representing the models at different thresholds (n) and the y-axis showing the MCC of our method minus the MCC of the best-performing model among other models. (TIF) [file pcbi.1012389.s013.tif]

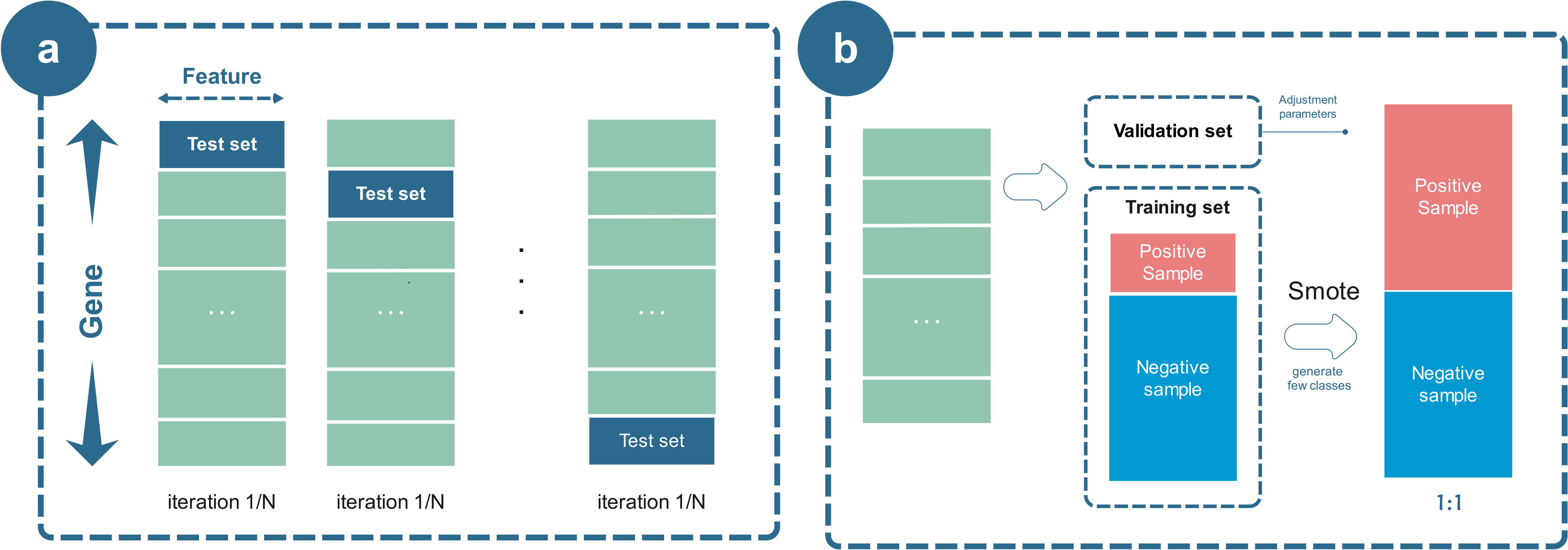

Supplement: S7 Fig — (a) Diagram of data partitioning for K-fold cross-validation. (b) Diagram of model training and validation for each fold. (TIF) [file pcbi.1012389.s014.tif]
