# Supplementary material for: IMI-driver: Integrating multi-level gene networks and multi-omics for cancer driver gene identification
Source: PLoS Comput Biol. 2024 Aug 26;20(8):e1012389. doi: 10.1371/journal.pcbi.1012389 (PMC11379397; doi:10.1371/journal.pcbi.1012389)
Supplement: Attachment — Submitted filename: response letter_v4.pdf [file pcbi.1012389.s022.pdf]

Dear Editor and Reviewers:

Thank you for reviewing our paper "IMI-driver: integrating multi-level gene networks and multi-omics for cancer driver gene identification" (Manuscript ID: PCOMPBIOL-D-24-00654) and providing valuable comments. We have revised and improved the paper according to your suggestions. We hope that this revised version meets your requirements and look forward to your reply.

The revised parts are highlighted in the manuscript (both highlighted version and clean version were uploaded). The responses to the referee's comments are as follows. We hope the new version can meet the requirements of the journal. Thanks for your kind help again.

Reviewer #1:

The identification of cancer driver genes is crucial for early detection, effective therapy, and precision medicine of cancer. This study introduces IMI-driver, a novel method that integrates multi-level gene networks and multi-omics data for the identification of cancer driver genes. The authors provide insights into the importance of integrating diverse biological networks (including common networks and tumor-specific networks) and multi-omics data for accurate cancer driver gene identification, and demonstrated the superior performance of IMI-driver compared to existing methods in predicting cancer driver genes.

While IMI-driver presents several strengths in integrating multi-level gene networks and multi-omics data for cancer driver gene identification, the manuscript also have some potential weaknesses need to be improved. My additional comments are as follows:

(1)In this paper, the authors used Top100 genes as the screening threshold. I don't think that's a fair way to compare different methods. As many methods do not use rankings as the screening threshold.

Our Response: Thank you for your insightful comments.

In our manuscript, we used the Top 100 genes as the screening threshold because many cancer driver gene prediction methods employ a ranking strategy to define

driver genes. Meanwhile, your point is very valuable. To evaluate the effectiveness of our method, we conducted two additional experiments:

Besides using the Top 100, we also used the Top 200, Top 300, Top 400, and Top 500 as thresholds to compare the performance of our strategy with other methods. The results show that our strategy consistently outperforms other methods across different thresholds (Supplementary Fig. 7).

We also directly used the class labels predicted by the machine learning model instead of using top N to define driver genes. The results are shown in Figure R1. According to the results, IMI-driver (TH), which is the driver gene predicted by the model directly, outperform IMI-driver (the current version of the manuscript (top 100 as drivers)). In addition, both IMI-driver (TH) and IMI-driver perform better than the other published methods. Since many published methods currently use the top N to identify driver genes, we still use the top N to define driver genes in our manuscript to compared the performance of all the models.

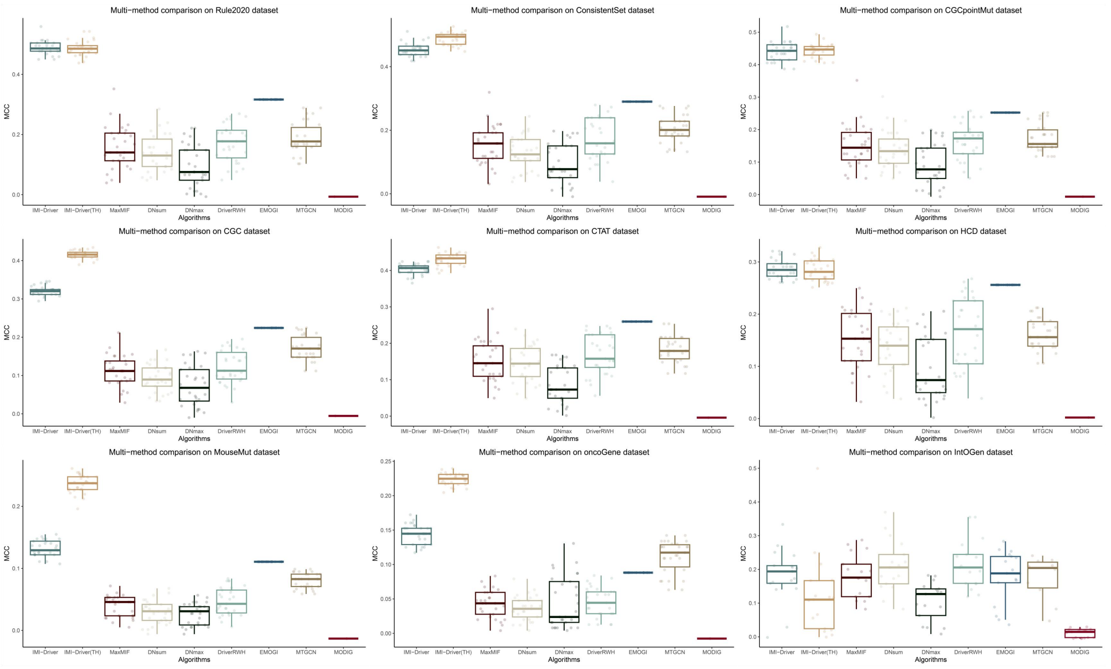

Figure R1. Comparison of the MCC of IMI-driver with other methods. Because we have added two new methods (the two methods you suggested: MODIG and MTGCN), the resulting now contain too many methods, making them difficult to read. Therefore, we only show the results of all network-based methods here.

(2)As the field of cancer driver gene identification is rapidly evolving, IMI-driver should compare with the latest state-of-the-art network-based methods, such as MODIG, MTGCN, MNGCL, and so on, to ensure its competitiveness and relevance in the current landscape.

Our Response: We sincerely appreciate your valuable feedback. Following your suggestion, we ran the first two methods you provided (MODIG and MTGCN) and compared the performance of all the methods. Since we compared numerous methods and the two newly added methods are both network-based strategies, for ease of reading, we included all network-based methods in Figure R1 (MCC) and Figure R2 (AUROC). According to the results (including Precision, F1-score and KS test p-value, data not shown for these three metrics), our method still outperforms the others , including the two newly added methods.

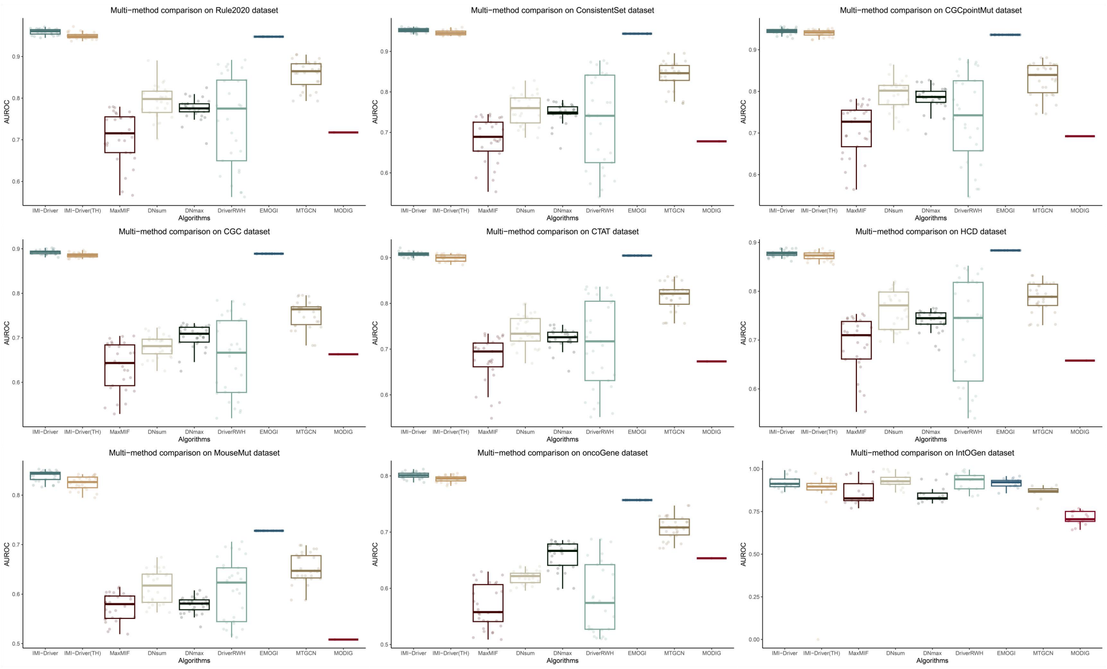

Figure R2. Comparison of the AUROC of IMI-driver with other methods.

(3)The interpretability of the results and the biological relevance of the identified novel driver genes could be further elaborated to provide a clearer understanding of the implications for cancer research and precision medicine.

Our Response: Thank you for your suggestion. In response to your feedback, we have provided more detailed biological explanations for these candidate genes (Line 217 -

242).

Further, we performed survival analyses based on TCGA database patient data and found statistically significant survival differences between the high and low expression groups for these six genes (two genes were marginal statistical significance) across multiple cancers. Supplementary Table 6 presents the results for all cancer datasets, while Figure 5 shows the results for each gene in a case dataset (Line 243 - 252).

(4)The figure legends are too simple in such as Fig. 2 and Supplementary Figures, which don't show the message clearly

Our Response: We apologize for the unclear description. They have now been corrected.

(5) The sample number of the multi-omics data of 29 cancer types from TCGA should be provided in the materials and methods, as well as the details resource or version of TCGA.

Our Response: Thank you for your valuable comments. We downloaded the standardized and processed TCGA dataset from the UCSC Xena database on July 28, 2022. Following your suggestion, we have included the omics data information and sample information used in this manuscript in Supplementary Note 1 and Supplementary Table 3.

Reviewer #2:

Considering that cancer is caused by the dysregulation of several genes at various levels of regulation, the authors present IMI-driver, a model that integrates multi-omics data into eight biological networks to predict cancer drivers. The experimental results confirm the effectiveness of this method. The major highlight of this work is the integration of different levels of biological networks, especially the incorporation of cancer-specific networks into the gene representation process, which has not been addressed in other methods. Therefore, I believe this work is quite innovative. Additionally, the manuscript is well-structured and written, and the results

are promising. However, some points need to be taken into consideration.

Major issues:

1.The authors define the top 100 genes as driver genes and define six new cancer driver genes by comparing them with known driver gene sets. Experimental findings suggest that these genes might indeed be potential driver genes. I am curious whether selecting the top 150 or 200 genes for each cancer type as driver genes would still yield potential driver genes. The authors should demonstrate this result to show the robustness of their method.

Our Response: Thank you for your suggestion. In our experiments, we evaluated genes ranked from the top 100 to the top 500, ultimately selecting the top 100 genes (For different thresholds, we also compared the performance of our method with other methods. The results show that our method outperforms the others across various thresholds (Supplementary Fig. 7).

Notably, among the top 200 genes (the result of top 150 is a subset of top 200), RAD51 and MITF are found to be driver candidates. Despite not appearing in the list of known driver genes or being predicted as potential driver genes by previous approaches, RAD51 and MITF exhibit significant differences between patients and normal individuals across multiple cancers, as detailed in Figure R3. In addition, The RAD51 gene family have crucial non-redundant roles in the repair of severe DNA damage related pathway (Cancer Letters. 2005 Mar 10; 19(2):125-135) and RAD51 is the target for anti-cancer therapy (Proceedings of the National Academy of Sciences. 2008; 105(52):20810-20815). In the meanwhile, MITFMITF are reported to be a tumor suppressor in non-small cell lung cancer (Aging (Albany NY). 2021 Jan 15; 13(1): 646–674). Furthermore, it is a crucial regulator of immunity, and it may be related to the resistance to anti-PD1 therapy (Experimental & Molecular Medicine. 2024; 56:311–318; Molecular Cancer. 2020; 19: 170).

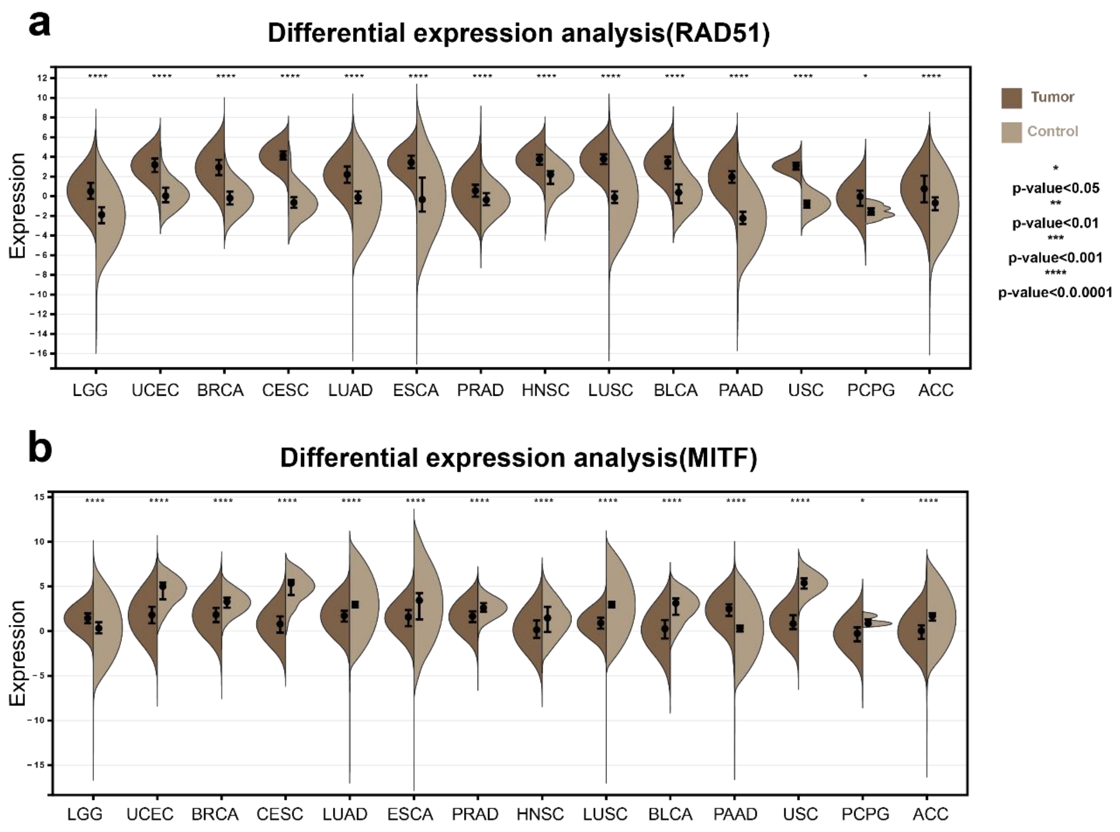

Figure R3. Differential expression analysis of RAD51 and MITF.

2.Regarding Figure 2, the evaluation metrics seem limited, and additional evaluation metrics may be needed.

Our Response: We apologize for any lack of clarity in our description. In our experiments, we selected Matthews Correlation Coefficient (MCC) as the primary evaluation metric (Fig. 2). Additionally, Area Under the Receiver Operating Characteristic Curve (AUROC), Precision, F1-score and the Kolmogorov-Smirnov (KS) test P-value were also used as evaluation metrics for our model. The detailed results for AUROC, Precision, F1-score and the Kolmogorov-Smirnov (KS) test P-value are provided in the Supplementary Fig. 2 - Supplementary Fig. 5. All these results show that our model perform the best.

3.Evaluate the computational efficiency of the IMI-driver model, particularly in terms of runtime and memory usage. Discuss strategies employed to optimize performance, such as parallel processing or algorithmic optimizations, and provide

recommendations for researchers seeking to apply the model to large-scale datasets.

Our Response: Thank you for your valuable feedback on the computational efficiency of our IMI driver model. We have carefully evaluated the performance of the model in terms of runtime and memory usage. We conducted algorithmic time testing on breast cancer, which has the largest sample size (1095 samples).

As shown in the table (Table R1), our method requires a significant amount of time due to the construction of multiple networks, multi-level network embedding calculations, and the final classifier prediction. Overall, the runtime decreases as the number of Threads increases. We recommend using 10 Threads (50 GB of memory), in which case our model takes approximately 22 hours to run. We have also mentioned this issue in the discussion section of the main text (Line 310 - Line 316).

Table R1. Runtime assessment of IMI-driver .

Number of Threads	Memory (G)	Time (Seconds)
5	25	1.34e05
10	50	8.00e04
15	75	7.22e04
20	100	6.94e04
25	125	6.73e04
30	150	6.58e04

4.Discuss avenues for refining the IMI-driver model, such as incorporating additional omics data modalities or exploring alternative network integration techniques. This would guide researchers in extending the proposed methodology and addressing remaining challenges in cancer genomics.

Our Response: We greatly appreciate the suggestions you provided for further enhancing our paper. Following your suggestion, we discussed the potential areas for expanding our model in the discussion section (Line 293 - Line 309). For your convenience, we have also included the relevant section here for your review:

In summary, IMI-Driver emerges as a robust and effective model for identifying

cancer driver genes, offering scalability to integrate diverse biological networks and datasets. Additionally, we provide a scalable research framework that constructs multi-level networks based on multi-omics data and represents the network nodes for classification. The technical details within the framework can be adjusted, and it can be applied to other biomedical fields beyond driver gene prediction. In this study, we used gene expression data, miRNA expression data, somatic mutation data, and methylation data. Other high-throughput sequencing data, such as proteomics data, can also be incorporated into our framework. At the network level, we integrated eight biological networks, including five cancer-specific networks and three common networks. Of course, other networks, such as metabolic networks, can also be included in the analysis in practical applications. In terms of multi-network embedding, besides the methods we used, other approaches suitable for multi-layer graph embedding can also be applied. For the final model prediction, in addition to driver gene prediction, theoretically, any other type of node prediction, such as drug target prediction, can also use our framework. In summary, we provide a scalable framework that can be used for various biomedical problems.

Minor issues:

1.Besides some conventional evaluation metrics, the authors also use the Ks-test to assess the performance of each method. However, the authors did not explain why the Ks-test can serve as an effective evaluation metric.

Our Response:

In previous studies, the KS test was used to assess whether a gene set is significantly enriched at the top of a ranked gene list (Science. 2006 Sep 29; 313(5795): 1929–35). Following this strategy, we used it to evaluate whether the list of driver genes is significantly enriched in our ranked gene list (where higher ranks indicate a higher likelihood of being predicted as driver genes by our model). In this context, a smaller p-value (from the KS test) indicates a higher degree of enrichment of true driver genes within the model-predicted gene list. Thank you for your suggestion; we have

added this part to Section 4.5.

2.The clarity of Figure 2 is insufficient, especially the text inside it is quite blurry, which affects its readability. I suggest the authors remake Figure 2.

Our Response: We apologize for any confusion. We have revised Figure 2 to enhance clarity and intuitiveness.

3.There are too few figures in the main text, and more details may need to be presented in the main text.

Our Response: Due to space constraints, we apologize for presenting only a selection of images in the original manuscript, with additional charts and graphs included in the Appendix. In this revised version, we have added Fig.4(a) and Fig.5 to enhance clarity.

4.Provide more detailed explanations of the IMI-driver model, including the specific algorithms utilized for multi-view collaborative network embedding and the integration of multi-omics data into biological networks.

Our Response: Thank you for your valuable comments on our paper. Following your suggestion, we have incorporated a detailed explanation of the IMI-driver model into the main text, focusing particularly on the multi-view collaborative network embedding algorithm (Section 4.3) and the integration of multi-omics data in biological networks (Section 4.2.3 and Section 4.2.4).

Reviewer #3: In this study, the authors propose IMI-driver, a model that integrates multi-omics data into eight biological networks and applies Multi-view Collaborative Network Embedding to embed the gene regulation information from the biological networks into a low dimensional vector space to identify cancer drivers. However, the

following issues need to be resolved by the authors before consideration for publication.

1. The authors should give a clear definition of what is cancer driver gene. Regarding the concept of a cancer driver gene, if the author's definition is solely based on genomic mutations, it raises the question of why it is necessary to integrate data from multiple omics. If, however, the impact of mutations on the system is considered, please provide specific experiments to demonstrate the extent of influence that driver mutations identified by IMI-driver methods have on cancer-related biological processes. If the scope of cancer driver genes extends beyond genomic mutations, why are comparative methods predominantly derived from driver mutation approaches? In summary, the fundamental issue is that the authors need to clarify their clear definition of driver gene in this paper, otherwise the various downstream evaluations are meaningless.

Our Response: Thank you for your detailed review and valuable comments on our paper. Based on your suggestions, we have added definitions of driver genes and described the necessity of using multi-omics data for driver gene prediction. The relevant changes can be found in the manuscript from Line 71 to Line 93.

(1) Definition of cancer driver genes. We appreciate your concern regarding the definition of cancer driver genes and would like to clarify further. As noted, driver genes encompass more than genomic mutations alone. Cancer development involves complex, multifactorial dysregulations across various cellular mechanisms (Bioinformatics 2022, 38:4901-07; British journal of cancer 2020, 122: 465–72; Cell 2017, 168 (4):629-643; Science 2013,339:1546–55). In our study, we used driver genes from multiple gold-standard databases, such as CGC (Nat Rev Cancer. 2018; 18: 696–705), to train and validate our model (K-fold cross validation).

(2) The need to integrate multi-omics data. Regarding the integration of multi-omics data, we believe cancer arises from dysregulation across multiple cellular mechanisms. These mechanisms, spanning gene expression, protein interactions, and epigenetic modifications, collectively influence cancer development. Relying solely on genomic mutation data may not fully elucidate these complexities. Thus, integrating

multi-omics data aids in comprehensively identifying potential driver genes, distinguishing them from passenger genes, and offering precise targets for cancer research (Nucleic Acids Research.2024 Jan 5; 52(D1): D1246-D1252).

(3) Impact of driver genes on biological processes. We totally agree with you that the importance of demonstrating the effects of driver genes on cancer-related biological processes. In response, we conducted differential expression (Line 212 - 216) and survival analyses on the identified driver genes to highlight their significant impact (Line 243 - 252). While specific biological experiments are crucial for further validation, which we acknowledge as a limitation in our study, we substantiate our findings by referencing existing literature that corroborates the functions and mechanisms of these genes in carcinogenesis (Line 217 - 242). Moreover, we performed pathway enrichment analysis (DAVID: https://david.ncifcrf.gov/summary.jsp) on the top 100 genes predicted by our method. The top 10 pathways are listed in Table R2. The results show that these genes are enriched in cancer-related pathways, with the 'Pathway in cancer' ranking first.

Table R2. Functional annotation of the top 100 genes predicted by our method

Pathways	P-value	FDR
hsa05200:Pathways in cancer	3.13E-33	3.06E-31
hsa05215:Prostate cancer	4.88E-16	1.24E-14
hsa01521:EGFR tyrosine kinase inhibitor resistance	4.95E-16	1.24E-14
hsa05206:MicroRNAs in cancer	5.05E-16	1.24E-14
hsa05224:Breast cancer	1.43E-15	2.80E-14
hsa05230:Central carbon metabolism in cancer	2.51E-15	4.10E-14
hsa01522:Endocrine resistance	1.44E-14	2.01E-13
hsa05225:Hepatocellular carcinoma	1.66E-14	2.03E-13
hsa05226:Gastric cancer	3.10E-14	3.37E-13
hsa05218:Melanoma	1.07E-13	9.57E-13

We hope these clarifications address your concerns adequately.

2 A single gene usually have multiple methylation sites, and similarly, a gene has multiple sites and types of mutations. Please elucidate specifically how the

co-methylation network and co-mutation network are constructed.

Our Response: Thank you for your review and valuable comments. We apologize for not describing this clearly. In the new version, we have included the data processing details in the first paragraph of Supplementary Note 1.

All the TCGA data sets were downloaded from UCSC Xena (July 28, 2022). For the gene expression data, miRNA expression data and mutation data, the high-level processed version (i.e. level 3) were used directly. In this context, the data have already been processed (by UCSC Xena) to use genes as the basic units. For example, gene expression values have been processed to reflect the value for each gene, and somatic mutation data have been processed to indicate whether a somatic mutation exists for each gene.

For the DNA methylation value, we focused on DNA methylation sites within promoter regions. Given that each gene can have multiple methylation sites, we selected all sites within the promoter region of each gene. We computed the median methylation level of these sites to represent the overall methylation level of each gene. These median values were then used to assess correlations between gene pairs to construct the DNA methylation interaction network.

For the co-mutations network, we used somatic mutation data, details regarding the construction of the DNA methylation interaction network and co-mutations network are provided in Sections 4.2.3 (Co-expression network and co-methylation network) and 4.2.4 (Co-mutations network) of the main text.

3. The authors should provide detailed information on the process of obtaining network-specific embeddings for each gene. Is this a supervised or unsupervised learning approach? If it's the former, how is the label for each gene determined?

Our Response: Thank you for your comment. In our study, we used the MANE approach to integrate multiple biological networks to obtain an embedded representation of each gene, a process that is based on an unsupervised learning approach. The final step, which involves using XGBoost to predict driver genes, is supervised learning. A detailed description is included in our revised manuscript (Section 4.3).

Reviewer #4:

****Identification of cancer driver genes holds significant importance in precision medicine research. With the advent of high-throughput data, leveraging multi-omics data for cancer driver gene prediction has gained momentum. Cancer originates from the dysregulation of several genes across various regulatory levels, yet current methods fail to address this. This paper proposes eight biological networks to capture gene regulatory relationships at different levels, represents genes via network embedding, and utilizes XG-boost for driver gene prediction. Experimental results demonstrate the method's effectiveness, supported by ablation studies and case analyses. I believe the presented approach is a promising solution for driver gene prediction, exhibiting noteworthy innovation, significant workload, and well-structured content. However, I have the following questions:

(1) As described above, the paper's greatest contribution lies in the application of a gene representation method based on multiple biological networks. Therefore, I am curious about how the performance would be if other classifiers were used instead of XG Boost. The authors could consider discussing the impact of classifier selection on IMI-driver in the main text.

Our Response: Thank you for your valuable comments and suggestions on our paper. We have taken your feedback seriously and conducted an extensive study to compare the performance of IMI-driver using different classifiers. Following your recommendation, we employed a variety of classifiers including Support Vector Machine (SVM), Random Forest, Logistic Regression, and Naive Bayes. These experiments were conducted on the same dataset, and we evaluated their performance using metrics such as Matthews Correlation Coefficient (MCC), Area Under the Receiver Operating Characteristic Curve (AUROC), F1 score, Precision, and Kolmogorov-Smirnov (KS) test. Detailed results are presented in Table R3. The results show that all these models have relatively good classification performance, especially Logistic Regression, which, as a very simple classifier model, only

performs slightly worse than XGBoost. This indicates the effectiveness of our model framework. Incorporating your suggestions, we have discussed these results in the new version of the manuscript (Lines 283 - 292).

Table R3. Performance comparison of different classifiers.

Classifier	MCC	AUROC	F1	Precision	KS test (-log10(P-value))
XGBoost	0.334	0.897	0.645	0.559	124.049
Logistic Regression	0.319	0.884	0.638	0.538	113.654
Naïve Bayes	0.069	0.833	0.528	0.130	84.063
Random Forest	0.292	0.884	0.627	0.490	115.555
SVM	0.201	0.746	0.588	0.331	43.874

The metric is the average value of the method across nine gold-standard datasets.

(2) In the case study section, the authors introduce six new candidate driver genes. I am curious about the prediction results of some well-known cancer driver genes, such as TP53, KRAS, BRCA1, etc., in the model. This result could also reflect the value of the model, and the authors should describe it in the paper.

Our Response: Thank you for reviewing our paper and providing valuable suggestions. We appreciate your emphasis on the importance of predicting well-known cancer driver genes (e.g., TP53, KRAS, BRCA1, etc.). Demonstrating the prediction results of these genes not only showcases the effectiveness of our proposed model compared to existing research but also enhances the practical applicability and credibility of our work in the field of biological network embedding and driver gene prediction.

In the previous version of the manuscript, we did not discuss the prediction of these well-known cancer genes by our model. Based on your suggestion, we have added Fig 4a. As shown in the figure, BRCA1, BRCA2, KRAS, and TP53BP1 are all predicted by our method to be part of the driver gene sets in various cancers. This demonstrates that our method can indeed predict well-known cancer driver genes (in the new version of the paper, we have included these results in Lines 205 - 208).

In addition, we have detailed the prediction results of these well-known driver genes. Specifically, among the 17 cancers (with benchmark information from IntOGen)

analyzed:

1) *TP53 was predicted as a driver gene in 15 cancers from the IntOGen collection of specific driver genes. It ranked in the top 100 in 2 cancers and in the top 200 in 5 cancers.*
2) *KRAS was identified as a driver gene in 10 cancers, ranking in the top 100 in 9 cancers and in the top 200 in 13 cancers.*
3) *BRCA1 was labeled as a driver gene in 1 cancer according to the IntOGen-specific driver gene collection, ranking in the top 100 in 8 cancers and in the top 200 in 10 cancers.*

Detailed results for other genes can be found in Table R4.

Table R4. Times of the Known driver genes to be predicted as top N across all the cancers.

Gene	Top 100	Top 200	Top 300	Top 500	Top 1000
TP53	2	5	6	8	10
KRAS	9	13	14	14	14
BRCA1	8	10	11	11	12
BRCA2	6	10	13	13	14
EGFR	9	10	11	12	13
PIK3CA	13	14	14	14	14
BRAF	9	14	14	14	14
APC	8	10	12	13	15

(3) In addition to leveraging multi-omics data and biological networks, the authors have utilized features from previous studies (DROGE) for data augmentation. The DORGR features seem crucial in enhancing the model's performance. I am curious to know how the performance of XG-boost would be if only DROGR features were used for classification.

Our Response: Thank you for your suggestion. We conducted experiments to analyze the performance of XGBoost using only DROGR features, comparing it with IMI-driver (multi-omics and multi-layer network embedding feature). The results show that although DORGE has a certain level of performance, our IMI-driver significantly outperforms the model that uses only DORGE as the feature. This

demonstrates the effectiveness of our method (Detailed results can be found in Table R5).

Table R5. Comparison with DROGE.

Classifier	MCC	AUROC	F1	Precision	KS P-value (-log10 transformed)
IMI-driver	0.334	0.897	0.645	0.559	124.049
XGBoost+DROGE	0.138	0.798	0.541	0.383	107.678

The metric is the average value of the method across nine gold-standard datasets.

(4) In the ablation experiments section, why did the authors primarily focus on demonstrating the model's performance on the IntOGen database instead of other gold-standard datasets?

Our Response: Apologies for any confusion caused.

In this study, we used a total of nine gold standard datasets, among which only IntOGen provided tumor-specific driver gene data. Therefore, in our ablation experiments, as depicted in Figure 3.c, we conducted analyses on all gold standard datasets, as described in the article. Additionally, we performed ablation experiments specifically on IntOGen, a cancer-specific gold standard dataset, as illustrated in Figure 3.d. In the new version of the paper, we have clarified this section (Section 2.3).

(5) There is some overlap in content between the main text and the supplementary notes, such as the introduction of gold-standard datasets. The authors should remove these redundancies, for instance, the introduction of gold-standard datasets is found in both the "Data Collection" section of the main text and Supplementary Note 1.

Our Response: We appreciate your observation regarding the overlap in content between the main text and the supplementary notes, particularly in the introduction of the gold standard dataset. In our revised manuscript, we have remove this overlap to eliminate redundancies and ensure the paper's consistency and flow.

(6) In the background section, some recent studies on cancer driver gene research and deep learning, i.e., PMID: 37076339, PMID: 36161334, PMID: 35997565, PMID: 37843994, PMID: 37402191, PMID: 38485768., should be referenced to broaden the scope of the research.

Our Response: Thank you for your suggestion. We have cited these papers as reference [4][7][11][12][13][14] in the revised manuscript.

(7) More attention should be paid to the formatting of the references. For instance, references [32] and [33] have different formats, which need to be corrected.

Our Response: Thank you for your review and detailed feedback on our paper. We appreciate your observation regarding the issues with reference formatting, particularly the inconsistency noted with references [32] and [33]. In our revised manuscript, we have thoroughly check and standardize the formatting of all references to ensure compliance with journal specifications and requirements.

Thank you again for your valuable comments and suggestions on our paper.
